# Alpha Suppression Is Associated with the Tip-of-the-Tongue (TOT) State Whereas Alpha Expression Is Associated with Knowing That One Does Not Know

**DOI:** 10.3390/jintelligence10040121

**Published:** 2022-12-08

**Authors:** Edmund Qian-Long Shen, David Friedman, Paul Alexander Bloom, Janet Metcalfe

**Affiliations:** Department of Psychology, Columbia University, New York, NY 10027, USA

**Keywords:** tip-of-the-tongue (TOT), curiosity, alpha, alpha suppression, spectral analysis, ERP, consciousness, metacognition

## Abstract

The tip-of-the-tongue (TOT) state is a spontaneously occurring metacognitive state that indicates that the answer to a query is almost, but not quite, at hand, i.e., that resolution is imminent. Since the time of William James, a distinctive feeling of nagging frustration has been observed to be associated with TOT states. On a more positive note, TOT states are also associated with intense goal-directed curiosity and with a strong desire to know that translates into successful mental action. The present study showed that prior to the presentation of resolving feedback to verbal queries—if the individual was in a TOT state—alpha suppression was in evidence in the EEG. This alpha suppression appears to be a marker of a spontaneously occurring, conscious, and highly motivating goal-directed internal metacognitive state. At the same time, alpha expression in the same time period was associated with the feeling of not knowing, indicating a more discursive state. Both alpha and alpha suppression were observed broadly across centro-parietal scalp electrodes and disappeared immediately upon presentation of the resolving feedback. Analyses indicated that the occurrence of alpha suppression was associated with participants’ verbal affirmations of being in a TOT state, which is also related to subsequent expression of a late positivity when feedback is provided, and to enhanced memory.

## 1. Introduction

In their seminal article on meta-reasoning, Ackerman and Thompson (2017) proposed that when faced with a query or problem, the first metacognition that comes into play consists of a higher order evaluation or feeling which they call a ‘judgment of solvability’. This metacognition, they note, may trigger the impulse to actively engage in the effort needed to achieve the goal/answer. The experience, as well as the neural correlates of this initial metacognitive state, judgment, feeling, intuition, or appraisal—this second order ‘hunch’ that we may be able to come up with the answer—are the focus of the present article. 

As noted by Polanyi (1974): “This question goes back to antiquity. Plato set it in the Meno. He said that if we know the solution of a problem, there is no problem—and if we don’t know the solution, we do not know what we are looking for and cannot expect to find anything … The problem is ineluctable and can be answered only by recognizing a kind of intuition”. Polanyi goes on, somewhat poetically: “I have spoken of our powers to perceive a coherence bearing on reality, with its yet hidden future manifestations, the kind of foreknowledge we call a [solvable] problem. And we know that the scientist produces problems, has hunches, and, elated by these anticipations, pursues the quest that should fulfil these anticipations … Think of Einstein, when as a boy he came across the speculative dilemma of a light source pursuing its own ray... Through years of sometimes despairing enquiry, he kept up his conviction that the discovery he was seeking was within his ultimate reach and that it would prove worth the torment of its pursuit; and again Einstein proved right... The scientist is faced with similar decisions. An intuition which would merely point him out problems could not tell him which problem to choose. He must be able to estimate the gap separating him from discovery and he must also be able roughly to assess whether the importance of a possible discovery would warrant the investment of the powers and resources needed for its pursuit. Without this strategic intuition, he would waste his opportunities on wild goose chases and be soon out of a job” (p. 249). 

We propose that this potentially highly motivating metacognition that is experienced in at least some cases before serious attempts at solution begin, is, essentially, a judgment that the gap is not too great. Indeed, the experience that the answer that is sought is knowable by the problem solver, that it is within reach, or, indeed, that it is ‘almost known’ fuels its pursuit. Motivation is increased by the metacognitive feeling that the solver ‘almost knows’ the answer (Berlyne 1954), or that is within their Region of Proximal Learning (Metcalfe and Kornell 2005; Metcalfe et al. 2020; Metcalfe 2021). Metcalfe and Jacobs (2021) have called this kind of goal-directed motivation to know ‘Curiosity1.’ (Note that they also posited that there is a conceptually different kind of curiosity that they called ‘Curiosity2’. This latter is discursive and exploratory, and, according to Jacobs and Metcalfe (forthcoming) may also come into play, but in a different way, during problem solving.) Although highly salient Curiosity1-type feelings of imminence (Smith 1994) may not occur in all cases in which a person is provided with a solvable problem to solve, of course, when such metacognitive feelings do occur, they can be highly motivating (Schwartz and Metcalfe 2011). The strongest and most well studied case of ‘almost knowing’ is the tip-of-the-tongue (TOT) state, a distinctive metacognitive experience that intrudes upon people’s consciousness spontaneously.

It *is* something, in the Nagelian sense that it is something to be a bat (Nagel 1974, i.e., it is conscious), to be in a TOT state (Schwartz et al. 2000): it is a conscious state. As Carruthers (1989) has argued, much—indeed, perhaps most—of the time people are in states that do not involve consciousness. Furthermore, even metacognition can happen unconsciously. It has been argued (Schwartz and Metcalfe 2011) that conscious metacognition does not run continuously: metacognitive states, including feelings of knowing, judgments of learning, or confidence evaluations, do not impinge themselves upon the subject most of the time. Indeed, while many metacognitive judgments—judgments about one’s confidence in some answer, say, about whether one has understood, or about whether one is making progress—can be voluntarily accessed on demand, they do not routinely surface into consciousness spontaneously (Schwartz and Metcalfe 2011) without being solicited. TOT experiences are the exception. They occur with an immediacy in awareness rivaling visual, auditory, or pain qualia, and they are compelling to mental action. 

There are many reports of the phenomenological ‘feel’ of TOT states, notably the famous quotation of William James (1890, p. 251) who said: “The state of our consciousness is peculiar. There is a gap therein; but no mere gap. It is a gap that is intensely active. A sort of wraith of the name is in it, beckoning us in a given direction, making us at moments tingle with the sense of our closeness and then letting us sink back without the longed-for term. If wrong names are proposed to us, this singularly definite gap acts immediately so as to negate them. They do not fit into its mold. And the gap of one word does not feel like the gap of another, all empty of content as both might seem necessarily to be when described as gaps … ” In another example, Brown and McNeill (1966) observed that: “The signs of it [a TOT] were unmistakable; [the subject] would appear to be in mild torment, something like the brink of a sneeze, and if he found the word, his relief was considerable” (p. 326). The TOT state, then, appears to be experientially distinctive and not particularly pleasant. 

Schwartz (1999) has argued that this state of consciousness is universally experienced, as evidenced by the existence of a linguistic expression denoting the state in all 51 languages that he investigated. An exception is Q’eqchi’, a language that functionally has no written communications (although there is a written guide, there are no books, newspapers or other widespread written communication in Q’eqchi’) and has no distinct expression for the TOT state. Nevertheless, Brennen et al. (2007) showed that speakers of Q’eqchi’ immediately understand the phenomenology of TOTs. They both claimed to experience such a state, and, when given trivia questions such as, “Who is the President of the United States?”, “Who is Argentina’s most famous ever footballer?”, or “Who is the indigenous Guatemalan from Quiché who won the Nobel Peace Prize in 1992?^1^” they indicated that although they could not say the name now, they knew that they would remember it later, and expressed the kinds of typical frustration associated with being in a TOT state. They appeared to have the subjective, phenomenological experience. American Sign Language, too, has an expression for this state, according to Schwartz (1999), although it is tip-of-the-fingers-related rather than being tongue-related. 

Furthermore, the TOT state has been documented to have functional significance: it acts as a trigger to mental action. It is highly motivationally relevant (Ryan et al. 1982) and is associated with goal-directed Curiosity1 (Bloom et al. 2018; Brooks et al. 2021; Fastrich et al. 2018; Loewenstein 1994; Metcalfe et al. 2017; Metcalfe and Jacobs 2021). Indeed, the TOT state has been taken as the quintessential exemplar of the feeling state underpinning people’s compelling goal-directed need to know. Metcalfe et al. (2017) showed that people were approximately twice as curious to know answers to questions when they were in a TOT state as compared to when they were not. Furthermore, when curious, information is better learned (Gruber et al. 2014; Kang et al. 2009; Marvin et al. 2020; Murayama et al. 2019; Xu and Metcalfe 2016) and serves as a reward (FitzGibbon et al. 2021). Cleary et al. (2021) have shown that when they were in TOT states, participants were more willing to accept the risk of taking a potentially penalizing test, and Lau et al. (2020) have shown that, when curious, people are willing to endure adverse circumstances including even the possibility of electric shock, in order to obtain the answer. 

The TOT state, then, is one of the most widely studied of metacognitive states (e.g., Bock 1987; Brown 2012; Brown and McNeill 1966; Funnell et al. 1996; Gardiner et al. 1973; Heine et al. 1999; Koriat and Lieblich 1974, 1977; Perfect and Hanley 1992; Schwartz 1999; Schwartz and Cleary 2016; Schwartz and Metcalfe 2014; Schwartz and Pournaghdali 2020). It has implications for our understanding of many cognitive processes including reasoning, problem solving, lexical access, and psycholinguistic processes (e.g., Bowers et al. 1990; Burke et al. 1991; Gollan and Brown 2006; Miozzo and Caramazza 1997; Nordmann et al. 2013); it presages changes in cognition with normal aging (MacKay and Burke 1990; Schwartz and Frazier 2005); it is implicated in decision-making and perceptual biasing (Cleary 2019; Cleary and Claxton 2015), and it is well known in investigations of memory retrieval (Gollan and Brown 2006; Koriat 2000; Schwartz 2008; Schwartz and Smith 1997). 

Some neural correlates of the TOT state have been investigated: fMRI studies have pointed to activation of several brain regions including the anterior cingulate cortex, dorsolateral prefrontal cortex, and inferior frontal cortex (Kikyo et al. 2001; Maril et al. 2001, 2005). In addition, people show an increased late positivity in the ERP record starting at around 250 ms after receiving informational feedback when they had been in a TOT state as compared to when they had not been in a TOT state (Bloom et al. 2018; and see Diaz et al. 2007; Galdo-Alvarez et al. 2009). 

As has often been noted, the TOT state (and curiosity, along with it, see Metcalfe et al. 2022, Experiment 2) is resolved immediately upon definitively attaining or being given the answer. Once one knows for certain that one has the answer, the nagging phenomenological state disappears (Loewenstein 2007). Thus, there should be rapid changes in mental and brain processes with different neural involvement implicated pre- and post-feedback. The neural processes involved upon resolution, presumably, should be different from those prior to resolution. Observations of such rapid state changes are more detectable with EEG than they are with fMRI. 

Ryals et al. (2021) have shown that a finely modulated pupillary dilation is observable while the person is in the TOT state. This finding suggests that a particular state of conscious awareness—that we hypothesized might be obvious in EEG spectral analysis insofar as brain oscillations are often thought to reflect feeling states—may accompany affirmations of being in a TOT state. We have been able to find only one previous EEG spectral study of the TOT state (Resnik et al. 2014). The 10 participants in that study were of mean age 70.2 years. While it is well known that the TOT state is much more frequent in older than younger adults, it is not known whether the mental processes might also differ. Our study used college students. Our materials were also different: the previous study used a pictorial face-naming task whereas our study used general information queries. 

Our primary interest in the present paper, then, was in investigating potential EEG neural correlates of the TOT state itself, prior to its resolution, in order to obtain further evidence regarding this state of conscious awareness. While previous work has identified spectral EEG correlates of various metacognitive processes (Jin et al. 2019; Klimesch 1997, 2012), only a few studies have investigated oscillatory brain signals specifically associated with the TOT state (Resnik et al. 2014). Because relatively little work has been conducted in this area, we did not have specific a priori hypotheses concerning particular frequency bands or electrodes. Thus, we took an exploratory approach towards identifying such correlates by conducting a broad search for spectral EEG measures associated with the experience of TOT states. As these analyses identified differences in the alpha frequency band (8–12 Hz), we provide an overview of the alpha suppression literature and offer post hoc explanations for these findings in the Discussion.

We were primarily interested in investigating neural correlates of the TOT state *prior to* its resolution; that is, the markers of the actual state, rather than the consequences of having been in a TOT state previously. However, we also sought to compare these contemporaneous markers to those that ensued upon resolution of the TOT state; that is, once feedback was provided. Accordingly, we analyzed data that had been collected in our lab (Bloom et al. 2018) but had not previously been examined regarding the neural correlates of the TOT state itself. Here, we will present some post-feedback results, but will primarily examine the pre-feedback spectral correlates of TOTs—in short, we will focus on the TOT state itself. 

## 2. Methods 

### 2.1. Participants

Participants were 30 healthy adults studying at Columbia University with ages ranging from 18 to 32 years old (16 male, 13 female, 1 did not report gender), with a mean age of 21.27 years and SD of 4.26 years. Participants were recruited through flyers in buildings on the Columbia University campus and emails to lists of previous study participants who had consented to being contacted. All participants were fluent in English, and reported being right-handed and free of medications known to impact the central nervous system. All participants provided written informed consent, and were compensated at a rate of USD 15/hour. All protocols were approved by the Columbia University and New York State Psychiatric Institute institutional review boards. 

One participant did not report experience of any TOT states, and was thus excluded from all analyses. To ensure an adequate numbers of trials, the EEG recordings were examined to determine whether each participant had at least 5 usable trials (see EEG preprocessing) for both the tip-of-the-tongue (TOT) and no-TOT conditions. Three subjects failed this criterion by having less than 5 usable TOT trials, and were thus excluded during preprocessing. A final total of 26 participants were included in all EEG analyses. 

### 2.2. Design

The study was a within-participants quasi-experimental design. All participants were read a set of general information questions during the initial question period. After being asked each question, they were given 3 s to try to answer, in the pre-feedback period. All responses in which people provided the correct answer were excluded. If they had not given the answer within the 3 s pre-feedback interval, they then made a TOT judgment, which was followed by a 500 ms fixation period after which correct answer feedback was given. They were then given 1 s to process the feedback. Once all questions had been presented, participants were given a surprise retest on all questions. Question order was randomized for each participant. EEG was recorded during the pre-feedback and the post-feedback phases (see EEG procedures below). In the analyses, below, the answer to the TOT probe was treated as if it were an independent variable with two levels (TOT vs. no-TOT). 

### 2.3. Stimuli

The stimuli were 150 general information questions taken from Nelson and Narens (1980), and updated and corrected by Bloom et al. (2018). For example, one question was “What is the capital of Jamaica?” (Answer: Kingston). All correct answers were a single word. The questions were displayed with PsychoPy2 in a large white font with a gray background on a computer monitor (Peirce 2007) for 3000 ms. Answers were displayed for 1000 ms. All stimuli and experiment PsychoPy code are available at https://osf.io/nkh9u/ (accessed on 8 May 2021).

### 2.4. Procedure

First, EEG caps were applied. Next, participants were told that they would be given a series of questions to try to answer verbally and, if they could not answer within the given time, to report whether or not they were in a TOT state. Participants were also instructed that they would get to view the answers to each question for which they could not generate an answer within the allotted time. Before starting the study, an experimenter explained the TOT state as a phenomenon in which “you feel sure you know the answer and think you can get it—it is imminent—but you cannot think of it at the moment.” Participants completed one practice trial prior to beginning the experiment to ensure that they understood the instructions and were responding appropriately.

**Figure 1:**
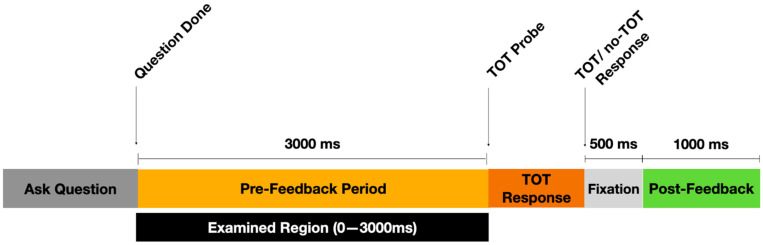
Timeline of individual trials. Note: ‘Ask Question’ represents a window of variable length when the experimenter was reading aloud the general information question to the subject. ‘Examined Region’ represents the 3000 ms window—corresponding to the pre-feedback/subject response period—that was selected for spectral and time-frequency analysis.

As is shown in Figure 1, during each trial, the question text appeared onscreen and was read aloud by an experimenter sitting next to the participant in the EEG booth. Upon finishing reading the question, the experimenter pressed a key on a keyboard to start the 3 s pre-feedback response period. If a verbal answer was given by the participant during this period (whether correct or not), the experimenter pressed a key to move directly to the next trial (i.e., the next question) without providing the answer to the question. If the participant did not give a response they were prompted to indicate whether they were experiencing a TOT state or not (yes/no). The experimenter recorded their responses with a keyboard press, which triggered a 500 ms period with a fixation cross on the screen, followed by a 1000 ms period during which the feedback (correct answer) displayed. Following feedback, a blank screen was shown for 750–1250 ms (jittered), before the next trial began. All participants completed 150 trials. 

Following the TOT/feedback phase, experimenters removed the EEG cap, and participants were given a 2 min break. Next, participants were told that there would be a test phase, and were given instructions on how to complete the test. The participant left the EEG booth for the final test phase during which he or she saw the same 150 questions in a different randomized order, and were allowed to type in their answer. They were told to give their best guess in response to each question even if they were not sure of the answer. No feedback was provided during this phase. After the test phase, participants filled out a demographics questionnaire, and were debriefed, compensated, and were allowed to view the answers to all of the general information questions if they desired. 

### 2.5. Response Scoring 

Participants’ final recall responses were evaluated against the correct answers using an approximate pattern matching algorithm (AGREP, generalized Levenshtein edit distance = .1), and a naive experimenter checked all of the algorithmically-based scoring. The final recall results were reported in Bloom et al. (2018). 

### 2.6. Electroencephalographic (EEG) Recording and Processing

Brain electrical activity was recorded during the TOT and feedback phases from 62 scalp sites (sintered Ag/AgCl) mounted in an Electrocap (Neuromedical Supplies). The EEG data were digitized at 500 Hz (DC; high-frequency cutoff of 100 Hz; right-forehead ground). Electrodes were placed on the outer canthus of each eye to record horizontal eye movements, and directly above and below the left eye for vertical movements. Activity was originally referenced to the nose and re-referenced offline to the average of the left and right mastoids. Impedances were maintained below 10 kΩ throughout the experiment. Prior to analyses, all recordings were filtered using a 0.1–25 Hz IIR-Butterworth bandpass filter to remove DC drift and muscle movements. Bad channels and data periods were systematically removed using the EEGLAB artifact subspace reconstruction (ASR) algorithm, with rejection criteria (flatline rejection threshold = 5 s, high-frequency noise threshold = 4 SD, minimum acceptable correlation = 0.7) as recommended by Delorme’s group-analysis preprocessing pipeline (Delorme and Makeig 2004; Kothe et al. 2019). Interpolation for removed channels was done across all trials. Independent component analysis (ICA) decomposition was conducted with the Picard algorithm, and artifactual components were labeled and rejected via ICLabel (rejection probability threshold = 0.90) (Ablin et al. 2018). As no feedback was presented for trials in which participants verbally recalled the answers during the 3 s response period after question presentation, these trials were not included in the EEG analyses. Only trials in which no answer was given and participants were either in a TOT or no-TOT state were included in analyses. 

To address the pre-feedback time period, the EEG data were analyzed during the 3000 ms answering period after the experimenter had finished asking the general information question and initiated (via button press) the onset of the EEG epoch. This time period included mental activity that was presumably associated with attempts at determining the answer to the question and, importantly, the TOT or no-TOT mindset. The examination region was defined as the 3000 ms between the ‘question-done’ key-press and the TOT probe because this was the timespan with the cleanest resolution, reflecting primarily internal cognitive phenomena rather than responses to external stimuli. Since the experimenter was talking (asking the question aloud) during the 1 s before the question-done trigger, that period was filled with a high amount of literal noise. Those auditory stimuli would translate into substantial EEG noise, rendering it difficult if not impossible to parse whether ERP/ event-related spectral perturbations (ERSP) phenomena observed during that window were due to sound of the experimenter’s voice as compared to the participant’s cognitive state. The 3000 ms of silence between the question-done trigger and the subsequent TOT probe was thus the cleanest window for epoching. To address the post-feedback time period, the EEG data were analyzed during the 1000 ms after feedback was displayed. 

Trials in which participants provided the wrong answer were excluded because of the problem of variable epoch length. An incorrect answer could have been given several hundred milliseconds after the question-done trigger in some trials, and nearly 3 s after the trigger in other trials. Such a wide range of response times is incompatible with ERP and ERSP analyses, which require consistent epoch durations in order to generate averaged curves across participants. Comparing TOT trials with ‘don’t know’ trials is thus the cleanest design for an EEG-based study of this particular phenomenon, insofar as the two trial types have consistent, stable epochs which can be meaningfully compared with one another. 

### 2.7. Spectral Analyses

Overall spectral power of the 3000 ms pre-feedback and 1000 ms post-feedback periods was calculated using the EEGLAB *std spec* algorithm, applying fast-Fourier transforms (FFTs) to each trial. Log power at each frequency was calculated and expressed in microvolts squared (µV^2^). After performing FFTs for all individual electrodes, the spectral decompositions of the channels were then aggregated to generate the averaged scalp topography over a specified frequency range—given our focus on alpha power differences, we computed averaged topographies specifically for spectral power in the 8 Hz to 12 Hz band.

ERSPs were computed for the 3000 ms pre-feedback period using the EEGLAB *newtimef* algorithm. In accordance with typical *newtimef* parameters, the number of wavelet cycles was set to increase linearly with frequency by a factor of 0.8, beginning at the lower-bound frequency of 3 Hz. Time-frequency images were time-locked to the question-done cue, as is shown in Figure 1, and power in decibels (dB) over the 3000 ms window was computed with the subtraction of a 500 ms pre-question-done baseline. Time-frequency images averaged over electrodes identified to be significant in the scalp topographies were then computed for the TOT and no-TOT conditions.

## 3. Results

### 3.1. Full-Period Spectral Decompositions

#### 3.1.1. Individual-Electrode Analysis

**Figure 2:**
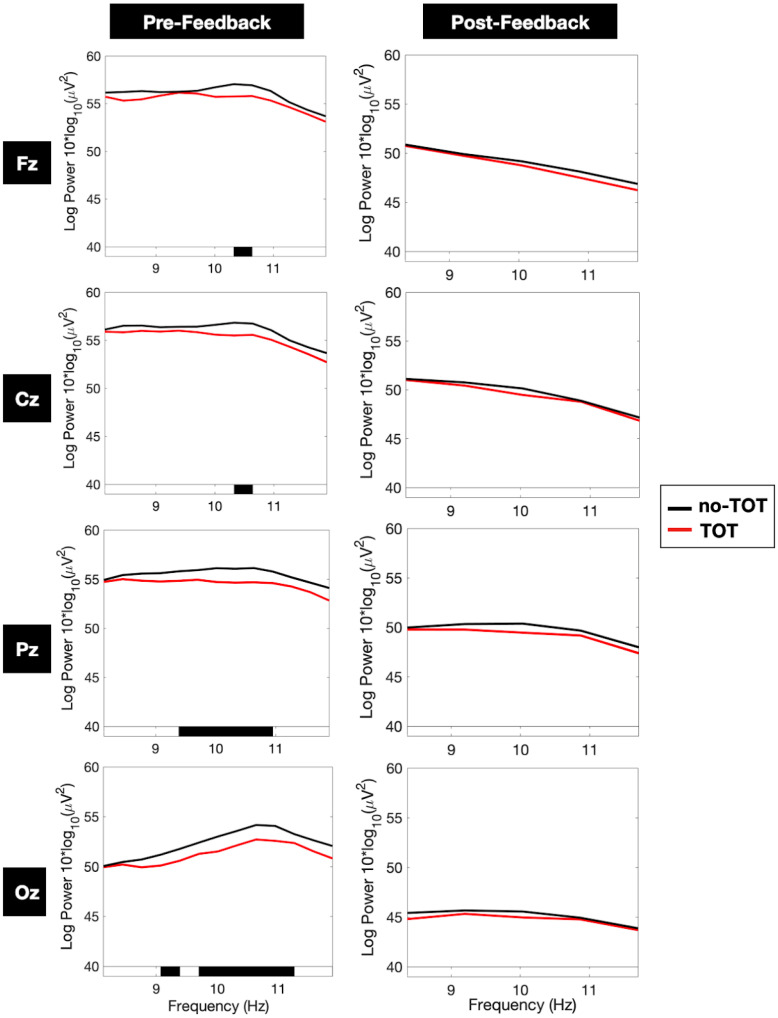
Frequency-power plots of four main midline electrodes during the pre-feedback and post-feedback periods. Note: ‘pre-feedback’ and ‘post-feedback’ refer to the 3000 ms and 1000 ms windows depicted in Figure 1. Fz, Cz, Pz, and Oz denominate the frontal, central parietal, and occipital electrodes placed along the midline of the participant’s scalp. Black bars under each plot represent frequency banks where the difference between the TOT and no-TOT conditions was statistically significant at the 1% level after applying the Bonferroni multiple-comparisons correction.

As is shown in Figure 2, Column A, examination of the main midline electrodes (Fz, Cz, Pz, Oz) revealed that the TOT state was associated with lower alpha spectral power during the 3000 ms pre-feedback window. All four main midline electrodes included regions of alpha power—particularly in the 10 Hz to 11 Hz range—where differences between the TOT and no-TOT conditions were significant (*p* < 0.01) after application of the stringent Bonferroni multiple-comparisons correction. Alpha spectral differences between the two conditions were also more pronounced towards the back of the head, with broader bands of significance in the parietal (9.5–11 Hz) and occipital (9–11 Hz) electrodes than the frontal and central electrodes (10.3–10.6 Hz).

No statistically significant spectral differences were observed during the 1000 ms post-feedback window, as shown in Figure 2, Column B. Alpha power was not significantly different when participants were receiving the correct answer, regardless of whether they had been in a TOT state or not. 

#### 3.1.2. Averaged Scalp Topographies

**Figure 3:**
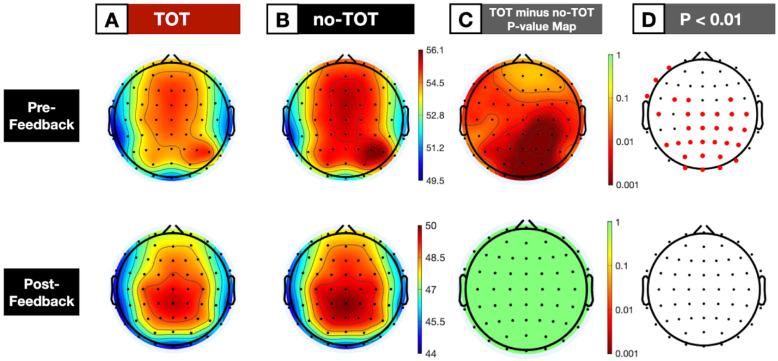
Whole-scalp averaged spectral topography for TOT versus no-TOT trials. Note: Columns (**A**,**B**) depict the topography of log power over the alpha band (8–12 Hz) for TOT and no-TOT conditions, respectively. Pre-feedback power was based on a spectral analysis of the 3000 ms examined period after the question-done cue. Post-feedback power was based on a spectral analysis of the 1000 ms period after the 500 ms fixation cross that indicated the onset of correct answer feedback. Column (**C**) represents the Bonferroni-corrected *p* values for differences between TOT and no-TOT trials at each point across the scalp. Column (**D**) highlights in red the electrodes where difference in TOT versus no-TOT was significant at the 1% level after Bonferroni multiple-comparisons correction.

Scalp topographies over all 62 electrode sites of aggregate log power in the alpha band (8–12 Hz) supported the results of the individual-electrode analyses. As is shown in Figure 3, in the pre-feedback window, alpha power was significantly lower for TOT trials than no-TOT trials after Bonferroni correction. TOT-associated alpha suppression was stronger over the posterior regions of the scalp, and skewed slightly toward the right hemisphere, as is shown in Figure 3, Columns C and D. Thirty-one electrodes on the scalp—primarily those on the central, parietal, and occipital regions—were significant at the 1% level for lower alpha power in the TOT state, as Figure 3, Column D shows. No differences were observed for topographies of delta (0.5–4 Hz), theta (4–7 Hz), or beta (13–30 Hz) waves over the same 3000 ms window. 

As is shown in the lower portion of Figure 3, Column C, no significant spectral differences were observed for TOT versus no-TOT trials during the post-feedback period. Bonferroni-corrected *p*-value maps indicated that there were no significant differences in alpha power over the scalp during the time period in which participants were receiving the correct answer; that is, in the 1000 ms window after making their TOT/no-TOT response. No individual electrodes were significant at the 1% level for differences in aggregate spectral power on the 8–12 Hz band (see, Figure 3, lower portion of Column D). In addition, no differences were observed for delta, theta, or beta wave topographies over the same window. 

As was the case with the frequency-power analyses of the main midline electrodes, whole-scalp topographies identified TOT-associated alpha suppression specifically in the 3000 ms pre-feedback window, when the question was unresolved and the participant was cogitating about a potential response. 

#### 3.1.3. ERSP Analysis

An averaged time-frequency image of the significant electrodes that are shown in Figure 3 (AF7, F7, FC1, FC3, FC4, FT7, Cz, C1, C2, C4, C5, C6, CPz, CP1, CP2, CP4, CP6, Pz, P1, P2, P3, P4, P5, P6, POz, PO3, PO4, PO8, Oz, O1, O2) was computed to examine how alpha power evolved over time in the pre-feedback window. (A similar analysis was conducted over the 9 central-parietal electrodes that were previously-reported to be significant in post-feedback TOT-associated activation in Bloom et al. (2018). This alternative analysis yielded highly similar results to those presented here.)

**Figure 4:**
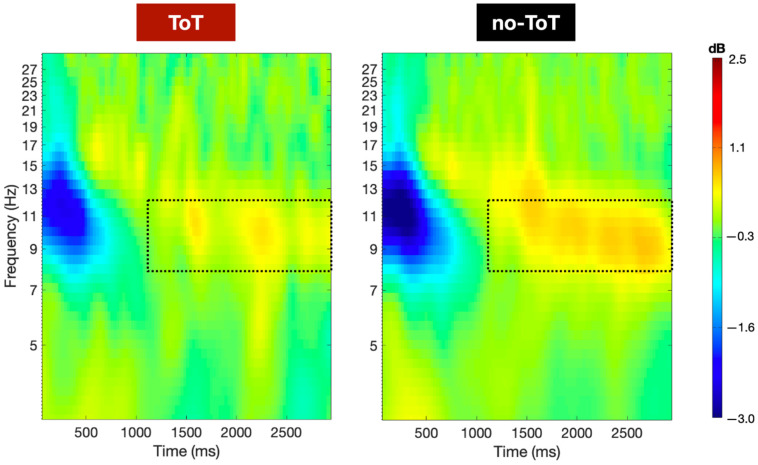
Time-frequency decomposition of spectral power during the pre-feedback period for the significant electrodes. Note: Plots represent the averaged time-frequency decompositions of the 31 electrodes which were found to have Bonferroni-corrected *p* < 0.01 significant differences in alpha power over the pre-feedback window: AF7, F7, FC1, FC3, FC4, FT7, Cz, C1, C2, C4, C5, C6, CPz, CP1, CP2, CP4, CP6, Pz, P1, P2, P3, P4, P5, P6, POz, PO3, PO4, PO8, Oz, O1, O2. The dotted box represents a window of particular interest (1000–3000 ms) in the alpha band (8–12 Hz), where higher consistent levels of alpha power were present in the no-TOT state relative to the TOT state.

As is shown in Figure 4, alpha power was low in the first 1000 ms (−3.0 dB) of the pre-feedback window relative to the 500 ms baseline period of comparison, for both the TOT and no-TOT conditions. Past 1000 ms, however, consistent alpha power was observed in the no-TOT ERSP image. While both the TOT and no-TOT images demonstrated some degree of alpha power in the 1000 ms to 3000 ms window, the no-TOT image reveals continuous perturbations of +0.6 to +0.8 dB in the alpha band relative to baseline. By contrast, the TOT image demonstrates less consistent alpha power, with perturbations primarily between +0.1 dB to +0.5 dB and only several plotted pixels reaching +0.7 dB. The given ERSP images thus provide a more granular portrait of the alpha differences noted in Figure 2 and Figure 3. Differences in alpha power in the TOT and no-TOT states appear primarily to be the result of a divergence that occurred at approximately 1000 ms after the question-done marker, with more consistent and higher-magnitude alpha-range power in the no-TOT state.

### 3.2. Event-Related Potential Analyses

**Figure 5:**
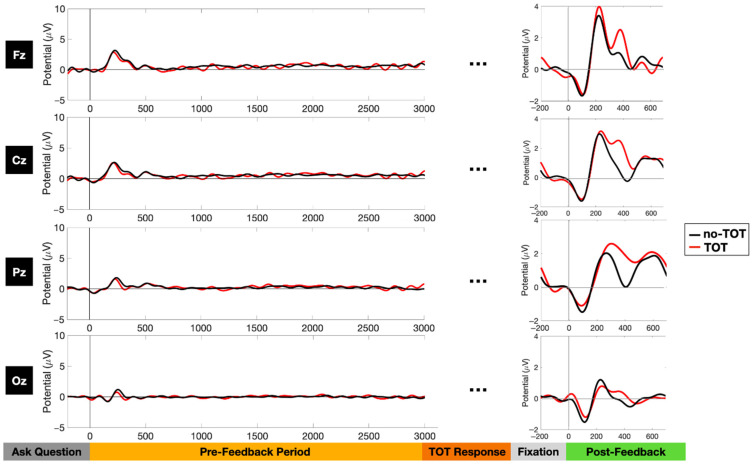
ERP plots of four main midline electrodes during the pre-feedback and feedback periods. Note: Fz, Cz, Pz, and Oz designate the frontal, central parietal, and occipital electrodes placed along the midline of the participant’s scalp. The pre-feedback ERPs were time-locked to the question-done cue, as illustrated in Figure 1. The post-feedback ERPs were time-locked to the start of the feedback window following a 500 ms fixation cross, also as illustrated in Figure 1. The post-feedback results are re-analyses, with a more stringent preprocessing algorithm, of results presented in Bloom et al. (2018). Ellipses (...) represent windows in the TOT response and fixation periods for which ERPs were not computed.

We also conducted an analysis of the ERPs, time locked to the offset of the question (the question-done signal), and a reanalysis^2^ of the ERPs following the correct answer feedback, as shown in Figure 5. This analysis revealed no differences between the TOT and the no-TOT conditions in the pre-feedback interval. Indeed, the analysis showed virtually no deviation from baseline. The right side of Figure 5 also shows our post-feedback reanalysis of the data that were previously presented in Bloom et al. (2018), using the updated preprocessing pipeline described above. Despite the more stringent rejection criteria of the current processing, we still observed greater post-feedback positivity over the central and parietal electrodes for TOT trials compared to no-TOT trials. 

The current combination of ERSP and ERP data thus indicate that TOT in the pre-feedback window is associated with substantial spectral differences (alpha suppression) but no ERP differences, while having been in a TOT state prior to feedback results in changes in the post-feedback window that are characterized by ERP differences but no spectral differences. 

## 4. Discussion

The most important result presented here is the finding that just after people had been presented with a question, a neural correlate of what appeared to be a metacognitive evaluation of solvability—in the form of alpha or alpha suppression—was in evidence during the time period after which they had processed the question but had not yet discovered the answer. When people knew that they did not know, they showed a strong propensity toward alpha enhancement. When they were in a TOT state, however, alpha *suppression* was in evidence. This suppression occurred during the time period after which they had processed the question but had not yet discovered the answer. As Figure 2, Figure 3 and Figure 4 indicate, after about one second of consideration of the question, if people had not yet given an answer to the question, they either lapsed into alpha (when they knew that they did not know), or they exhibited alpha suppression (when they experienced a TOT). 

The spectral analysis also indicated that there were no other frequency bandwidths that were different when the person was in the TOT state as compared to the ‘don’t know’ state. Neither theta nor delta was in evidence, in either condition. Insofar as both of these are associated with sleep and also with deeply relaxed mental states, this is not surprising. It is more surprising that no gamma or beta were in evidence in either condition, insofar as these frequencies are associated with concentration, which might have been expected if people were actively trying to retrieve the answer to the question in the TOT state. In short, the only band of activation that was in evidence when people were trying to answer the question in the TOT and no-TOT states was between 8 and 12 HZ, and activation in this particular bandwidth was more prominent when people were in the don’t know state; it was suppressed when people indicated that they were in the TOT state. Interestingly, the only other study that used a spectral analysis to investigate TOTs (Resnik et al. 2014) also found alpha suppression. 

There was no significant difference between TOT and no-TOT conditions in the pre-feedback ERPs in our study. This is in contrast to a difference in pattern that was shown in an ERP study by Buján et al. (2012). Rather than presenting verbal questions, though, these authors provided pictures of faces for 1 s, then left a blank period of time for one second before asking subjects to indicate whether they knew the name, did not know the name, or thought they knew it but could not retrieve it—which, later, they classified as a TOT item if the person was also able to state the target’s profession, and choose the correct name in a 3-alternative forced choice test. They found that starting at around 800 ms after the onset of the picture of the face, the ERPs for items that were labelled known and not known converged and exhibited a higher positive amplitude than did those of the TOT items. The ERPs for TOT items diverged and tended toward the baseline. The divergence in the ERP tracings was quite distinctive by about 1350 ms. It is puzzling to us that what, purportedly, was increased processing in the TOT condition was marked by a decrease in amplitude—a return to baseline. In any event, the authors suggested that this ERP pattern indicted that “in TOT, the processing resources may still be involved in the search for lexical–phonological information about the person, reflecting a continued but unsuccessful effort in this category in order to resolve the conflict, whereas in know and in DK (don’t know) the processing resources may be completely released” (p. 989). 

The ERP differences between no-TOT and TOT items observed by Buján et al. (2012) prior to feedback were not found in our study. The most likely explanation for this difference between studies is that the famous face pictorial stimuli used in the Buján et al. (2012) experiment afforded a better alignment of the onset of processing than did the verbal queries that we had used. Our queries were of variable length and participants may have finished reading some of them before the experimenter had finished reading the question aloud and hit the button (the signal to which we aligned) indicating that presentation was complete. The explanation given by Buján et al. (2012) for their results, though, fits well with our spectral findings. They suggest that a short time after the question was presented, if the participant knew that they did not know the answer, their efforts to find the answer flagged. In our study, heightened alpha was exhibited at this point. For TOT items, though, people’s efforts toward solution likely persisted. This was exemplified, in our study, by alpha suppression.

ERP differences, reflecting differences in processing after the feedback was given, were in evidence in our study. There was an enhanced late positivity when people had been in a TOT state as compared to when they had not, and this late positivity, as well as their subjective claim about having been in a TOT state, was associated with enhanced recall (Bloom et al. 2018). We show these ERP results in Figure 5, using a slightly different preprocessing methodology. The spectral analysis of the post-feedback period, however, as is shown in the bottom panels of Figure 3, did not show any significant differences between TOT and no-TOT items. 

## 5. Conclusions

The primary result of interest in this study was that while increased alpha was related to the participant’s judgment that he or she did not know the answer to a query, alpha suppression was associated with their being in a TOT state. The TOT state is a well-known, tantalizing, spontaneously occurring, conscious, phenomenological state in which the participant feels like they ‘almost know’—a metacognitive marker that the answer is attainable. Our spectral analysis results are consistent with much research about the mental correlates of both alpha and alpha suppression as well as with the characteristics of TOT states. 

Spectral analysis has been extensively employed in studies investigating state differences in relation to attention. It has been widely acknowledged that both alpha expression and alpha suppression are functional correlates of brain activation, and the attendant mental processes (Klimesch 1999). Event-related synchronization of alpha band power (i.e., increases in alpha power) has often been interpreted as indicating reduced information processing in the underlying neuronal networks. It is associated with what some investigators call “cortical idling” and the corresponding mental idling (Pfurtscheller 1999). Suppression of alpha band activity is related to the increased excitability of neurons involved in implicated cortical areas, and has traditionally been related to enhanced attention and information processing (Neuper and Pfurtscheller 2001; Pfurtscheller and Lopes da Silva 1999). 

Consistent with this interpretation, mind wandering has been shown to be associated with high alpha power (e.g., Baldwin et al. 2017). Similarly, Arnau et al. (2020), using a mind-wandering probe task in synchrony with EEG, showed both that the primary task response accuracy was reduced when people were mind wandering, and that alpha power was increased when they were mind wandering. Like our own results, their effect was spatially widely distributed over the scalp. Thus, the increase in alpha power seen in trials in which people indicated that they have no idea of what the answer was, could be taken as a neural indicator that they had simply gone off task (though see below for an alternative interpretation). 

The finding of alpha suppression when the person was experiencing a TOT state suggests that rather than cognitively idling or mind wandering, participants were continuing to engage their full attention to the task at hand—trying to come up with the answer to the query. The tip-of-the-tongue state, of course, is well known to be highly cognitively engaging and attention focusing as the person attempts to mentally zero in on the specific required word that is the answer. 

Alpha suppression has been shown to be associated with convergent thinking, in general. It is notable that in TOT studies, the solution is singular—not any answer will do. It is also characterized as being the prototype of Curiosity 1, where Curiosity 1 is defined in terms of goal seeking (convergent) rather than goal avoidant (divergent), or of what computer scientists (see Wilson et al. 2014) call exploitation rather than exploration. Although perhaps the most parsimonious interpretation of our finding of high alpha when people do not know the answer is simply that they go off task, a second, and more charitable interpretation of the increase in alpha is also possible. It could be a reasonable strategy, in a circumstance in which one does not know the answer, to engage in divergent thinking. Indeed, Schooler (forthcoming) has argued that mind wandering is related to exploratory, divergent, creative thinking. If one fails to come up with the solution directly, then seeking the solution in a less focused manner—letting the mind wander elsewhere—could be strategic. In support of this possibility, a review by Fink and Benedek (2014), concluded that alpha power is related to creative thinking. For example, Jauk et al. (2012) manipulated the kind of thinking by instructing participants to solve an association task either by finding the most common solution (in the convergent condition) or the most uncommon solution (in the divergent condition). Raters assessed the answers given in the divergent condition as being more creative. The study found that alpha synchronization was associated with divergent thinking, whereas alpha suppression accompanied convergent thinking. 

A number of visual attention studies have shown that alpha suppression is associated with selective attention to a particular region in external visual space associated with an expectation or anticipation (triggered by a cue) that a target is about appear in that location. Bacigalupo and Luck (2019), for example, showed that a cortically circumscribed location, contralateral in the brain to the location of the expected appearance of the target, exhibited alpha suppression. In the TOT state, though, rather than attention being focused *outwardly*, in anticipation of an externally presented target, it, presumably is focused *inwardly* on the individual’s own mental events (see Cooper et al. 2003). In the case of TOT, alpha suppression seems to indicate an inwardly focused attentional expectation—it is a marker that the person, him/herself, is about to come up with the answer. Unfortunately, this expectation-of-an-internal-event-about-to-happen interpretation of our data is clouded by the fact that we actually presented, onscreen, the answer to every question as feedback 3 s after each question was posed. Accordingly, our result might have reflected external-solution anticipation rather than self-solution anticipation. Two considerations challenge this interpretation. First, if the alpha suppression that we observed occurred merely as an anticipation that the solution was about to be presented externally, it should have occurred regardless of whether the person was in the TOT state or not. However, it did not occur with no-TOT items. Second, the only other study that has looked at the spectral components of TOT (Resnik et al. 2014) did not present the solution externally as feedback (at least not until the end of the entire experiment, which was after a considerable temporal delay). Their study, like ours, showed alpha suppression when people were in the TOT state. That alpha suppression is a marker of an internally focused attentional state is consistent with both the decreased dual task performance (Ryan et al. 1982), and the increased pupillary responses (Ryals et al. 2021) that have been observed in conjunction with TOT states. 

The alpha suppression observed in our study was widely distributed over the scalp, as is shown in Figure 3. Interestingly, in the MEG TOT study on older adults (Resnik et al. 2014), alpha suppression was tightly localized to the left ventral temporal region. The reason for this difference in distribution is currently unknown, and requires further research. It may, of course, be attributable to differences in the populations (young versus older adults), or methods (picture naming versus general information). It is also possible, however, that it is due to the diversity of the content of what people were seeking when they were in the TOT states in the two experiments. The stimuli that were presented in the Resnik et al. (2014) study were much more uniform than were ours: they were always the names of famous people associated with the face pictures. Our answers, by contrast, traversed a large range of possible topics. It is possible that our distributed pattern occurred because of the diversity of the answers sought. We cannot, from the present data, distinguish among these possibilities. 

Other correlates of alpha suppression also line up with what is known about the TOT state. For example, the TOT state is associated with negative affect (Schwartz 1999). Interestingly, although the state itself may be experienced as frustrating (Schwartz et al. 2000), Cleary (2019) has found that items that are emotionally positively valanced are more likely to evoke TOTs. (It may be frustrating to be unable to remember the pleasurable target, whereas one may not care to remember a negatively valanced target and so the frustration of trying without immediate success is avoided.) Alpha suppression is associated with somewhat negative, rather than positive, affect. A highly focused but not especially pleasant state may be conducive to cognitive success. Kuhbandner et al. (2009, 2016), for instance, showed that people’s affective state influenced how easy it was for them to consciously perceive a masked target stimulus: a slightly negative affective state was more effective in facilitating perception. Furthermore, these authors, like others, also found that alpha was associated with the positive and alpha suppression with the negative affective state. 

Finally, to return to the relation of the TOT state to precognition and Meno’s paradox, as noted by Polanyi at the outset: it seems, at first blush, puzzling and perhaps even spooky that people might perceive a hidden reality and be motivated by such an intuition. In fact, though, this idea may not be so mystifying as it seems. People often have partial information about the answer. Indeed, many studies of TOTs show that people are aware of such partial information. They often know the first letter, the number of syllables, the gender, items that are semantically similar, and they often have access to knowledge that items that come to mind spuriously are incorrect (see Schwartz and Metcalfe 2011; Wade and Kidd 2019). Interestingly, an EEG study by Magazzini et al. (2016) linked partial awareness of words to alpha suppression in which the visual perception of the word is masked and sometimes participants can detect it and sometimes not. The authors observed alpha suppression in the partial-seeing case. 

High alpha power is associated with off-task thought, with mind wandering, and with divergent, creative thinking (e.g., Arnau et al. 2020; Bozhilova et al. 2020). It seems antithetical to the kind of focused determination that is called for if the individual knew that the answer was near and that he or she was hot on the trail. Yet this kind of divergent, playful thinking could be helpful if the individual had no sense that the answer was attainable and beckoning or even that the answer was blocked and that the trail had gone cold. Casting about, mind wandering, or even just relaxing and taking a deep breath, could be strategic when one knows that one does not know. This state does not typically exhibit anything like the intrusive phenomenological feel of the TOT state, which forces itself upon the consciousness of the subject. Indeed, alpha states are often characterized by a relaxed lack of self-awareness. 

By way of contrast, alpha suppression is associated with focused goal-directed attention: people are expectant that a solution will be forthcoming; they may have slightly negative disquieting feelings; they intuit partial information about the answer; they feel that it is knowable and even imminent. This latter metacognitive state that is associated with the TOT state and with alpha suppression (and that can be observed in the lab in a small way), may, when played out at scale in the real world, be the kind of ‘judgment of solvability’ that underlies the intuition that a scientific dilemma may be within the scientist’s ultimate reach and that it could prove worth the torment of its pursuit. 

## Data Availability

All stimuli and experiment PsychoPy code are available at https://osf.io/nkh9u/. EEG data are available by request from pab2163@columbia.edu.

## Notes

1 Maradona (or maybe Messi). Rigoberta Menchú, for her work for the rights of indigenous peoples and reconciliation between ethnic groups.

2 This post-feedback analysis was already reported, in detail, in Bloom et al. (2018). We reanalyzed their post-feedback data here because we used different preprocessing software for our other analyses and wanted consistency throughout.

## References

[B1-jintelligence-10-00121] Ablin Pierre, Cardoso Jean-François, Gramfort Alexandre (2018). Faster independent component analysis by preconditioning with Hessian approximations. IEEE Transactions on Signal Processing.

[B2-jintelligence-10-00121] Ackerman Rakefet, Thompson Valerie A. (2017). Meta-reasoning: Monitoring and control of thinking and reasoning. Trends in Cognitive Sciences.

[B3-jintelligence-10-00121] Arnau Stefan, Löffler Christoph, Rummel Jan, Hagemann Dirk, Wascher Edmund, Schubert Anna-Lena (2020). Intertrial alpha power indicates mind wandering. Psychophysiology.

[B4-jintelligence-10-00121] Bacigalupo Felix, Luck Steven J. (2019). Lateralized suppression of alpha-band EEG activity as a mechanism of target processing. The Journal of Neuroscience.

[B5-jintelligence-10-00121] Baldwin Carryl L., Roberts Daniel M., Barragan Daniela, Lee John D., Lerner Neil, Higgins James S. (2017). Detecting and quantifying mind wandering during simulated driving. Frontiers in Human Neuroscience.

[B6-jintelligence-10-00121] Berlyne Daniel E. (1954). A theory of human curiosity. British Journal of Psychology.

[B7-jintelligence-10-00121] Bloom Paul A., Friedman David, Xu Judy, Vuorre Matti, Metcalfe Janet (2018). Tip-of-the-tongue states predict enhanced feedback processing and subsequent memory. Consciousness and Cognition.

[B8-jintelligence-10-00121] Bock J. Kathryn, Ellis Albert (1987). Coordinating words and syntax in speech plans. Progress in the Psychology of Language.

[B9-jintelligence-10-00121] Bowers Kenneth S., Regehr Glenn, Balthazard Claude, Parker Kevin (1990). Intuition in the context of discovery. Cognitive Psychology.

[B10-jintelligence-10-00121] Bozhilova Natali, Cooper Ruth, Kuntsi Jonna, Asherson Philip, Michelini Giorgia (2020). Electrophysiological correlates of spontaneous mind wandering in attention-deficit/hyperactivity disorder. Behavioural Brain Research.

[B11-jintelligence-10-00121] Brennen Tim, Vikan Anne, Dybdahl Ragnhild (2007). Are tip-of-the-tongue states universal?. Evidence from an Unwritten Language Memory.

[B12-jintelligence-10-00121] Brooks Gregory, Yang Haopei, Köhler Stefan (2021). Feeling-of-knowing experiences breed curiosity. Memory.

[B13-jintelligence-10-00121] Brown Alan S. (2012). Tip of the Tongue State.

[B14-jintelligence-10-00121] Brown Roger, McNeill David (1966). The “tip of the tongue” phenomenon. Journal of Verbal Learning & Behavior.

[B15-jintelligence-10-00121] Buján Ana, Galdo-Álvarez Santiago, Lindín Mónica, Diaz Fernando (2012). An event-related potentials study of face naming: Evidence of phonological retrieval deficit in the tip-of-the-tongue state. Psychophysiology.

[B16-jintelligence-10-00121] Burke Deborah M., MacKay Donald G., Worthley Joanna S., Wade Elizabeth (1991). On the tip of the tongue: What causes word finding failures in young and older adults?. Journal of Memory and Language.

[B17-jintelligence-10-00121] Carruthers Peter (1989). Brute experience. The Journal of Philosophy.

[B18-jintelligence-10-00121] Cleary Anne M. (2019). The biasing nature of the tip-of-the-tongue experience: When decisions bask in the glow of the tip-of-the-tongue state. Journal of Experimental Psychology: General.

[B19-jintelligence-10-00121] Cleary Anne M., Claxton Alexander B. (2015). The tip-of-the-tongue heuristic: How tip-of-the-tongue states confer perceptibility on inaccessible words. Journal of Experimental Psychology: Learning, Memory, and Cognition.

[B20-jintelligence-10-00121] Cleary Anne M., McNeely-White Katherine L., Russell Shaylyn A., Huebert Andrew M., Hausman Hannah (2021). The tip-of-the-tongue state as a form of access to information: Use of tip-of-the-tongue states for strategic adaptive test-taking. Journal of Applied Research in Memory and Cognition.

[B21-jintelligence-10-00121] Cooper Nicholas R., Croft Rodney J., Dominey Samuel J., Burgess Adrian P., Gruzelier John H. (2003). Paradox lost? Exploring the role of alpha oscillations during externally vs. internally directed attention and the implications for idling and inhibition hypotheses. International Journal of Psychophysiology: Official Journal of the International Organization of Psychophysiology.

[B22-jintelligence-10-00121] Delorme Arnaud, Makeig Scott (2004). EEGLAB: An open source toolbox for analysis of single-trial EEG dynamics including independent component analysis. Journal of Neuroscience Methods.

[B23-jintelligence-10-00121] Diaz Fernando, Lindín Mónica, Galdo-Alvarez Santiago, Facal David, Juncos-Rabadán Onésimo (2007). An event-related potentials study of face identification and naming: The tip-of-the-tongue state. Psychophysiology.

[B24-jintelligence-10-00121] Fastrich Greta M., Kerr Tyson, Castel Alan D., Murayama Kou (2018). The role of interest in memory for trivia questions: An investigation with a large-scale database. Motivation Science.

[B25-jintelligence-10-00121] Fink Andreas, Benedek Mathias (2014). EEG alpha power and creative ideation. Neuroscience and Biobehavioral Reviews.

[B26-jintelligence-10-00121] FitzGibbon Lily, Lau Johnny King L., Murayama Kou (2021). The seductive lure of curiosity: Information as a motivationally salient reward. Current Opinion in Behavioral Sciences.

[B27-jintelligence-10-00121] Funnell Margaret, Metcalfe Janet, Tsapkini Kyrana, Reder Lynne M. (1996). In the mind but not in the tongue: Feeling of knowing in anomic patient H.W. Implicit Memory and Metacognition.

[B28-jintelligence-10-00121] Galdo-Alvarez Santiago, Lindín Mónica, Díaz Fernando (2009). The effect of age on event-related potentials (ERP) associated with face naming and the tip-of-the-tongue (TOT) state. Biological Psychology.

[B29-jintelligence-10-00121] Gardiner Fohn M., Craik Fergus I. M., Bleasdale Fraser A. (1973). Retrieval difficulty and subsequent recall. Memory & Cognition.

[B30-jintelligence-10-00121] Gollan Tamar H., Brown Alan S. (2006). From tip-of-the-tongue (TOT) data to theoretical implications in two steps: When more TOTs means better retrieval. Journal of Experimental Psychology: General.

[B31-jintelligence-10-00121] Gruber Matthias J., Gelman Bernard D., Ranganath Charan (2014). States of curiosity modulate hippocampus-dependent learning via the dopaminergic circuit. Neuron.

[B32-jintelligence-10-00121] Heine Marilyn K., Ober Beth A., Shenaut Gregory K. (1999). Naturally occurring and experimentally induced tip-of-the-tongue experiences in three adult age groups. Psychology and Aging.

[B33-jintelligence-10-00121] Jacobs William J., Metcalfe Janet, Smith Stephen M., Salvi Carola Salvi, Wiley Jennifer The role of Curiosity1 and Curiosity2 in the emergence of insight. The Emergence of Insight.

[B34-jintelligence-10-00121] James William (1890). The Principles of Psychology.

[B35-jintelligence-10-00121] Jauk Emanuel, Benedek Mathias, Neubauer Aljoscha C. (2012). Tackling creativity at its roots: Evidence for different patterns of EEG alpha activity related to convergent and divergent modes of task processing. International Journal of Psychophysiology.

[B36-jintelligence-10-00121] Jin Christina Yi, Borst Jelmer P., Van Vugt Marieke K. (2019). Predicting task-general mind-wandering with EEG. Cognitive, Affective, and Behavioral Neuroscience.

[B37-jintelligence-10-00121] Kang Min Jeong, Hsu Ming, Krajbich Ian M., Loewenstein George, McClure Samuel M., Wang Joseph Tao-yi, Camerer Colin F. (2009). The wick in the candle of learning: Epistemic curiosity activates reward circuitry and enhances memory. Psychological Science.

[B38-jintelligence-10-00121] Kikyo Hideyuki, Ohki Kenichi, Sekihara Kensuke (2001). Temporal characterization of memory retrieval processes: An fMRI study of the “tip of the tongue” phenomenon. European Journal of Neuroscience.

[B39-jintelligence-10-00121] Klimesch Wolfgang (1997). EEG-alpha rhythms and memory processes. International Journal of Psychophysiology.

[B40-jintelligence-10-00121] Klimesch Wolfgang (1999). EEG alpha and theta oscillations reflect cognitive and memory performance: A review and analysis. Brain Research Reviews.

[B41-jintelligence-10-00121] Klimesch Wolfgang (2012). Alpha-band oscillations, attention, and controlled access to stored information. Trends in Cognitive Sciences.

[B42-jintelligence-10-00121] Koriat Asher (2000). The feeling of knowing: Some metatheoretical implications for consciousness and control. Consciousness and Cognition.

[B43-jintelligence-10-00121] Koriat Asher, Lieblich Israel (1974). What does a person in a “TOT” state know that a person in a “don’t know” state doesn’t know. Memory & Cognition.

[B44-jintelligence-10-00121] Koriat Asher, Lieblich Israel (1977). A study of memory pointers. Acta Psychologica.

[B45-jintelligence-10-00121] Kothe Christian, Miyakoshi Makoto, Delorme Antoine (2019). clean_rawdata (Version 2.7) [Computer Software]. https://github.com/sccn/clean_rawdata/wiki.

[B46-jintelligence-10-00121] Kuhbandner Christof, Spachtholz Philipp, Pastötter Bernhard (2016). Bad things come easier to the mind but harder to the body: Evidence from brain oscillations. Cognitive Affective Behavioral Neuroscience.

[B47-jintelligence-10-00121] Kuhbandner Christof, Hanslmayr Simon, Maier Markus A., Pekrun Reinhard, Spitzer Bernhard, Pastötter Bernhard, Bäuml Karl-Heinz (2009). Effects of mood on the speed of conscious perception: Behavioural and electrophysiological evidence. Social Cognitive and Affective Neuroscience.

[B48-jintelligence-10-00121] Lau Johnny King L., Ozono Hiroki, Kuratomi Kei, Komiya Asuka, Murayama Kou (2020). Shared striatal activity in decisions to satisfy curiosity and hunger at the risk of electric shocks. Nature Human Behaviour.

[B49-jintelligence-10-00121] Loewenstein George (1994). The psychology of curiosity: A review and reinterpretation. Psychological Bulletin.

[B50-jintelligence-10-00121] Loewenstein George (2007). Exotic Preferences.

[B51-jintelligence-10-00121] MacKay Donald G., Burke Deborah M., Stone F. Gordon A., West Robert (1990). Cognition and Aging: A Theory of New Learning and the Use of Old Connections. Advances in Psychology.

[B52-jintelligence-10-00121] Magazzini Lorenzo, Ruhnau Philipp, Weisz Nathan (2016). Alpha suppression and connectivity modulations in left temporal and parietal cortices index partial awareness of words. NeuroImage.

[B53-jintelligence-10-00121] Maril Anat, Wagner Anthony D., Schacter Daniel L. (2001). On the tip of the tongue: An event-related fMRI study of semantic retrieval failure and cognitive conflict. Neuron.

[B54-jintelligence-10-00121] Maril Anat, Simons Jon S., Weaver Josh J., Schacter Daniel L. (2005). Graded recall success: An event-related fMRI comparison of tip of the tongue and feeling of knowing. NeuroImage.

[B55-jintelligence-10-00121] Marvin Caroline B., Tedeschi Ellen, Shohamy Daphna (2020). Curiosity as the impulse to know: Common behavioral and neural mechanisms underlying curiosity and impulsivity. Current Opinion in Behavioral Sciences.

[B56-jintelligence-10-00121] Metcalfe Janet, Overson Catherine E., Hakala Christopher M., Kordonowy Lauren L., Benassi Victor A. (2021). The Region of Proximal Learning and Curiosity. In Their Own Words: What Scholars Want You to Know about Why and How to Apply the Science of Learning in Your Academic Setting.

[B58-jintelligence-10-00121] Metcalfe Janet, Kornell Nate (2005). A Regional of Proximal Learning model of metacognitively guided study-time allocation. Journal of Memory and Language.

[B57-jintelligence-10-00121] Metcalfe Janet, Jacobs William J., Ball Linden J., Vallee-Tourangeau Frederic (2021). The two faces of curiosity in creative cognition: Curiosity1, Curiosity2 (and their interaction). International Handbook of Creative Cognition.

[B59-jintelligence-10-00121] Metcalfe Janet, Schwartz Bennett L., Bloom Paul A. (2017). The Tip-of-the-tongue (TOT) State and curiosity. Cognitive Research: Principles and Implications.

[B60-jintelligence-10-00121] Metcalfe Janet, Schwartz Bennett L., Eich Teal S. (2020). Epistemic curiosity in the Region of Proximal Learning. Current Opinion in Behavioral Sciences.

[B61-jintelligence-10-00121] Metcalfe Janet, Vuorre Matti, Towner Emily, Eich Teal S. (2022). Curiosity: The effects of feedback and confidence on the desire to know. Journal of Experimental Psychology: General. Advance online publication.

[B62-jintelligence-10-00121] Miozzo Michele, Caramazza Alfonso (1997). Retrieval of lexical–syntactic features in tip-of-the tongue states. Journal of Experimental Psychology: Learning, Memory, and Cognition.

[B63-jintelligence-10-00121] Murayama Kou, FitzGibbon Lily, Sakaki Michiko (2019). Process account of curiosity and interest: A reward learning model of knowledge acquisition. Educational Psychology Review.

[B64-jintelligence-10-00121] Nagel Thomas (1974). What is it like to be a bat?. The Philosophical Review.

[B65-jintelligence-10-00121] Nelson Thomas O., Narens Louis (1980). Norms of 300 general-information questions: Accuracy of recall, latency of recall, and feeling-of-knowing ratings. Journal of Verbal Learning and Verbal Behavior.

[B66-jintelligence-10-00121] Neuper Christa, Pfurtscheller Gert (2001). Event-related dynamics of cortical rhythms: Frequency-specific features and functional correlates. International Journal of Psychophysiology: Official Journal of the International Organization of Psychophysiology.

[B67-jintelligence-10-00121] Nordmann Emily, Cleland Alexandra A., Bull Rebecca (2013). Cat Got Your Tongue? Using the Tip-of-the- Tongue state to investigate fixed expressions. Cognitive Science.

[B68-jintelligence-10-00121] Peirce Jonathan W. (2007). PsychoPy—Psychophysics software in Python. Journal of Neuroscience Methods.

[B69-jintelligence-10-00121] Perfect Timothy J., Hanley J. Richard (1992). The tip-of-the-tongue phenomenon: Do experimenter-presented interlopers have any effect?. Cognition.

[B70-jintelligence-10-00121] Pfurtscheller Gert, Niedermeyer Ernst, Lopes da Silva F. H. (1999). EEG Event-Related Desynchronization (ERD) and Event-Related Synchronization (ERS). Electroencephalography: Basic Principles, Clinical Applications and Related Fields.

[B71-jintelligence-10-00121] Pfurtscheller Gert, Lopes da Silva F. H. (1999). Event-related EEG/MEG synchronization and desynchronization: Basic principles. Clinical Neurophysiology: Official Journal of the International Federation of Clinical Neurophysiology.

[B72-jintelligence-10-00121] Polanyi Michael, Cohen Robert S., Wartofsky Marx W. (1974). Genius in Science. Methodological and Historical Essays in the Natural and Social Sciences. Boston Studies in the Philosophy of Science.

[B73-jintelligence-10-00121] Resnik Karmen, Bradbury David, Barnes Gareth R., Leff Alex P. (2014). Between thought and expression, a magnetoencephalography study of the “tip-of-the-tongue” phenomenon. Journal of Cognitive Neuroscience.

[B74-jintelligence-10-00121] Ryals Anthony J., Kelly Megan E., Cleary Anne M. (2021). Increased pupil dilation during tip-of-the-tongue states. Consciousness and Cognition.

[B75-jintelligence-10-00121] Ryan Michael P., Petty C. Raymond, M Richard (1982). Motivated remembering efforts during tip-of-the-tongue states. Acta Psychologica.

[B76-jintelligence-10-00121] Schooler Jonathan, Smith Stephen M., Salvi Carola Salvi, Wiley Jennifer The case for curious mind wandering (mind wondering). The Emergence of Insight.

[B77-jintelligence-10-00121] Schwartz Bennett L. (1999). Sparkling at the end of the tongue: The etiology of tip-of-the-tongue phenomenology. Psychonomic Bulletin and Review.

[B78-jintelligence-10-00121] Schwartz Bennett L. (2008). Working memory load differentially affects tip-of-the-tongue states and feeling-of-knowing judgment. Memory & Cognition.

[B79-jintelligence-10-00121] Schwartz Bennett L., Pournaghdali Ali (2020). Tip of the tongue states: Past and future. Memory Quirks.

[B80-jintelligence-10-00121] Schwartz Bennett L., Cleary Anne M., Dunlosky John, Tauber Sean (2016). Tip-of-the-tongue states, déjà vu and other metacognitive oddities. Oxford Handbook of Metamemory.

[B81-jintelligence-10-00121] Schwartz Bennett L., Metcalfe Janet (2011). Tip-of-the-tongue (TOT) states: Retrieval, behavior, and experience. Memory & Cognition.

[B82-jintelligence-10-00121] Schwartz Bennett L., Metcalfe Janet, Schwartz Bennett L., Brown Alan S. (2014). Tip-of-the-tongue (TOT) states: Mechanisms and metacognitive control. Tip-of-the-Tongue States and Related Phenomena.

[B83-jintelligence-10-00121] Schwartz Bennett L., Frazier Leslie D. (2005). Tip-of-the-tongue states and aging: Contrasting psycholinguistic and metacognitive perspectives. Journal of General Psychology.

[B84-jintelligence-10-00121] Schwartz Bennett L., Smith Steven M. (1997). The Retrieval of Related Information Influences Tip-of- the-Tongue States. Journal of Memory and Language.

[B85-jintelligence-10-00121] Schwartz Bennett L., Travis Donald M., Castro Anthony M., Smith Steven M. (2000). The phenomenology of real and illusory tip-of-the-tongue states. Memory & Cognition.

[B86-jintelligence-10-00121] Smith Steven M., Metcalfe Janet, Shimamura Arthur P. (1994). Frustrated feelings of imminent recall: On the tip-of-the tongue. Metacognition: Knowing about Knowing.

[B87-jintelligence-10-00121] Wade Shirlene, Kidd Celeste (2019). The role of prior knowledge and curiosity in learning. Psychonomic Bulletin & Review.

[B88-jintelligence-10-00121] Wilson Robert C., Geana Andra, White John M., Ludvig Elliot A., Cohen Jonathan D. (2014). Humans use directed and random exploration to solve the explore–exploit dilemma. Journal of Experimental Psychology: General.

[B89-jintelligence-10-00121] Xu Judy, Metcalfe Janet (2016). Studying in the region of proximal learning reduces mind wandering. Memory & Cognition.

